# Macrocycles and Supramolecules as Antioxidants: Excellent Scaffolds for Development of Potential Therapeutic Agents

**DOI:** 10.3390/antiox9090859

**Published:** 2020-09-14

**Authors:** Jung-Seop Lee, In-ho Song, Pramod B. Shinde, Satish Balasaheb Nimse

**Affiliations:** 1 Institute of Applied Chemistry and Department of Chemistry, Hallym University, Chuncheon 200702, Korea; wndtjq93@hanmail.net (J.-S.L.); songin-ho@hallym.ac.kr (I.-h.S.); 2 Natural Products & Green Chemistry Division, CSIR-Central Salt and Marine Chemicals Research Institute (CSIR-CSMCRI), Academy of Scientific and Innovative Research (AcSIR), Council of Scientific and Industrial Research (CSIR), Bhavnagar 364002, Gujarat, India; pramodshinde@csmcri.res.in

**Keywords:** supramolecules, calix[n]arene, resorcinarene, calixtyrosol, calixpyrrole, cucurbit[n]uril, porphyrin, antioxidant, radical scavenger, oxidative stress, reactive oxygen species, reactive nitrogen species

## Abstract

Oxidative stress due to the high levels of reactive oxygen species (ROS) that damage biomolecules (lipids, proteins, DNA) results in acute inflammation. However, without proper intervention, acute inflammation progresses to chronic inflammation and then to several chronic diseases, including cancer, myocardial infarction, cardiovascular diseases, chronic inflammation, atherosclerosis, and more. There has been extensive research on the antioxidants of natural origin. However, there are myriad possibilities for the development of synthetic antioxidants for pharmacological applications. There is an increasing interest in the identification of novel synthetic antioxidants for the modulation of biochemical processes related to ROS. In this regard, derivatives of supramolecules, such as calix[n]arene, resorcinarene, calixtyrosol, calixpyrrole, cucurbit[n]uril, porphyrin etc. are gaining attention for their abilities to scavenge the free radicals. Supramolecular chemistry offers excellent scaffolds for the development of novel antioxidants that can be used to modulate free radical reactions and to improve the disorders related to oxidative stress. This review focuses on the interdisciplinary approach for the design and development of novel synthetic antioxidants based on supramolecular scaffolds, with potentially protective effects against oxidative stress.

## 1. Introduction

Oxidative stress, due to the high levels of reactive oxygen species (ROS) that damage biomolecules (lipids, proteins, DNA), eventually leads to many chronic diseases, including cancer [1,2], myocardial infarction [3,4], cardiovascular diseases (CVD) [5,6,7], chronic inflammation [8], atherosclerosis [9] and more [10,11]. Free radicals such as hydroxyl (OH^•^), superoxide (O^2–•^), and nitric monoxide (NO^•^) are the major ROS found in cells [12]. Antioxidants are the molecules that scavenge free radicals to combat cellular oxidative damage [13,14]. Antioxidants can slow or prevent the oxidation processes initiated by ROS that damages cells in the body [15]. Based on their activity, antioxidants fall into two categories: enzymatic and non-enzymatic antioxidants [16]. Enzymatic antioxidants break down and remove free radicals, while non-enzymatic antioxidants work by interrupting free radical chain reactions [17]. Antioxidants exert their effect via numerous mechanisms, including inhibition of the formation of free radicals by reducing hydroperoxides (ROO^•^) and hydrogen peroxides, the scavenging of active free radicals, sequestering metal ions and repairing and clearing oxidative damages [18]. Antioxidants, therefore, have tremendous applications in the pharmaceutical industry due to their prophylactic and therapeutic activities [19,20].

There is increasing interest in the identification of novel antioxidants for the modulation of biochemical processes related to ROS [21,22]. The use of antioxidants, including enzymes, natural products, and synthetic compounds, is a logical therapeutic intervention for many ROS-related diseases, including cancer [23,24]. Considerable attention has been given to the design and development of potent antioxidants and the determination of their radical scavenging ability in complex samples, including cell culture studies and in vivo models. However, the development of novel antioxidants with both high pharmacological activity and few side effects is a challenging field [25]. In this regard, the use of supramolecular scaffolds, one of the many approaches that have been used for the development of novel antioxidants, can prove fruitful in the coming years.

Research on supramolecules and their applications has gained considerable attention in the last decade. Several classes of supramolecules have been developed for various applications, including chemosensors, biosensors, drug-delivery vehicles, and prodrugs. However, their application in the development of potent antioxidants has yet to be explored to its full capacity. Hence, supramolecular scaffolds such as calix[n]arene, resorcinarene, calixtyrosol, calixpyrrole, cucurbit[n]uril, porphyrin etc. provide a novel platform to design and develop novel antioxidants with both high pharmacological activity and few side effects. The development of synthetic antioxidants is attractive, as it allows one to manipulate the physicochemical properties to improve the antioxidant properties for various applications. It is crucial to notice that there are several reports on the inclusion complexes of these macrocycles, including cyclodextrins with antioxidant activities [26,27,28,29,30]. However, this review is focused mainly on the direct chemical modifications of calix[n]arene, resorcinarene, calixtyrosol, calixpyrrole, cucurbit[n]uril, and porphyrin scaffolds to synthesize the novel antioxidant compounds.

**Figure 1:**
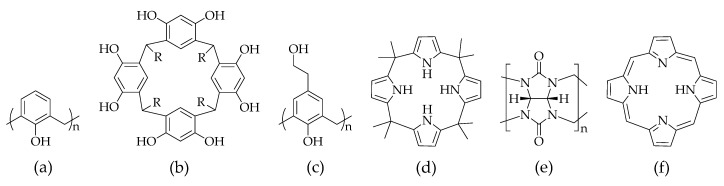
Supramolecular scaffolds used in the development of antioxidants (**a**) calix[n]arene; (**b**) resorcinarene; (**c**) calixtyrosol; (**d**) calixpyrrole; (**e**) cucurbit[n]uril; (**f**) porphyrin.

This review aims to explore the advances in the development of antioxidants, based on calix[n]arene, resorcinarene, calixtyrosol, calixpyrrole, cucurbit[n]uril, and porphyrin (Figure 1).

## 2. Supramolecular Scaffolds Used for the Development of Antioxidants

### 2.1. Calix[n]arene

**Figure 2:**
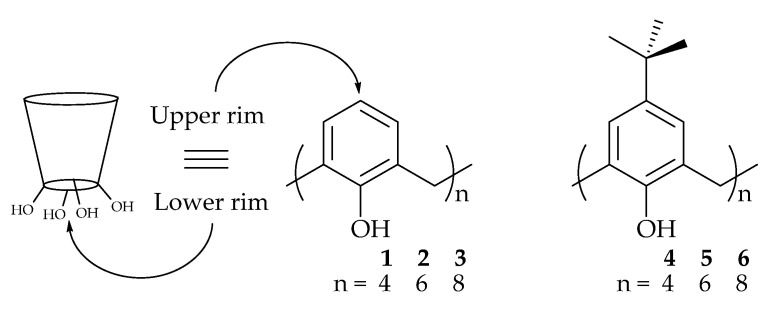
Calix[n]arene derivatives **1**–**6**.

Calixarenes are cyclic oligomers obtained by the pyrolysis of a polymer produced from the condensation of formaldehyde with *p*-alkylphenols in the presence of a strong base [31,32]. Depending upon the number of monomers, they can be classified as calix[4]arene 1, calix[6]arene 2, and calix[8]arene **3** (Figure 2). Calixarenes can be substituted with a variety of functional groups on their upper and lower rims, resulting in molecules with a variety of biological applications [33]. Water-soluble calix[4]arene derivatives have been exploited for their molecular recognition properties [34,35,36]. Since the calixarenes can be tailored to improve their water solubility, they can be excellent platforms for the development of useful antioxidants. Calixarenes are also known to poses antioxidant features for over a couple of decades [37,38].

**Scheme 1:**
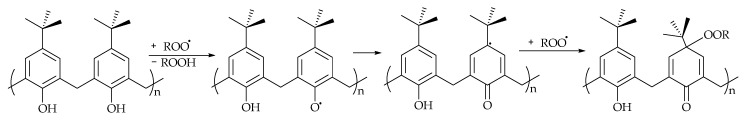
Mechanism of radical scavenging activity of t-butylcalix[n]arene derivatives.

Gorghiu et al. reported the contribution of *p*-*t*-butylcalix[n]arenes (**4**, **5** and **6**) (Figure 2) in the thermal stability of different types of low-density polyethylenes (LDPE) [39]. They found that the antioxidant feature of *p*-*t*-butylcalix[4]arene 4 is higher than the p-t-butylcalix[6]arene 5 and p-t-butylcalix[8]arene **6** for tested LDPE. The proposed mechanism is presented in Scheme 1.

Among the polyhydroxy phenolic compounds, the hydroxycinnamic acid derivatives are abundant in nature and are reported to possess strong antioxidant properties [40,41,42]. The amide derivatives of cinnamic acid are also reported to have significant radical scavenging ability [43]. Considering the potential of hydroxycinnamic acid (HA) as a potent antioxidant, Consoli et al. designed and synthesized the calixarene platform clustered with HA [44].

**Figure 3:**
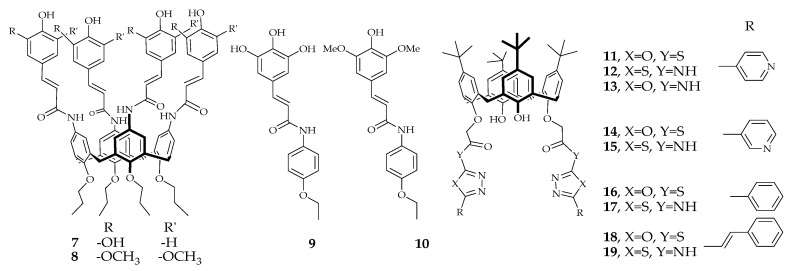
Structures of calix[4]arene derivatives **7**–**19**.

The radical scavenging and antioxidant activities determined by using 2,2-diphenyl-1-picrylhydrazyl radical (DPPH^•^) and AIBN^•^–induced linoleic acid peroxidation test showed that the compounds **7** and **8** showed enhanced activity as compared to the corresponding monomers **9** and **10** (Figure 3). The rate constant values (*k*_1_ > 1.5 × 10^4^) and the stoichiometric factors *n* (number of DPPH^•^ quenched/antioxidant molecule) for **7** (*n* = 7.7) and **8** (*n* = 2.7) indicated their strong radical quenching ability.

In 2012, Patel et al. reported the synthesis and evaluation of calix[4]arene based 1,3,4-oxadiazole and thiadiazole derivatives **11–19** (Figure 3) [45]. The conjugation of **4** (*tert*-butylcalix[4]arene) with 1,3,4-oxadiazole and 1,3,4-thiadiazole derivatives allowed them to synthesize novel antioxidant calixarene derivatives. All compounds showed significant DPPH^•^ scavenging activity. Notably, the compounds containing the oxadiazole ring were more potent antioxidants than other compounds. The DPPH^•^ scavenging activity of compound **18** (82.6%), containing oxadiazole ring and S bridge connecting to the calix[4]arene moiety, was comparable with the ascorbic acid (92.0%), which is used as a standard in the assays.

**Figure 4:**
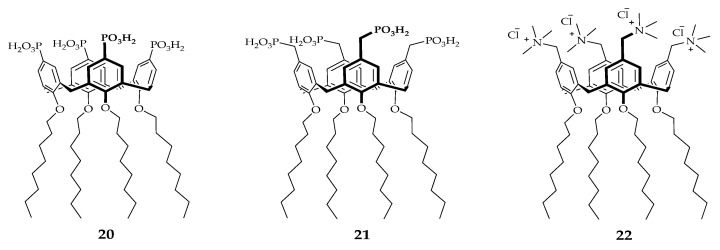
Structures of calix[4]arene derivatives **20**–**22**.

James et al. reported the synthesis and evaluation of antioxidant activities of amphiphilic phospholipid calix[4]arenes, that mimic the micellular delivery systems [46]. They evaluated the antioxidant activity compounds **20**, **21** and **22**, (Figure 4) using PC12 cells that were pre-treated with these compounds and then stressed with either H_2_O_2_, menadione, or glutamate. The obtained results indicated that the PC12 cells treated compounds **20**, **21** and **22** showed significant inhibition in ROS generation than the untreated control groups. Furthermore, the loading of curcumin in the amphiphiles of these compounds not only increases the delivery of curcumin to the cytoplasm, but also enhances the antioxidant capacity. The results establish that calix[n]arenes are potentially useful synthetic antioxidants.

**Scheme 2:**
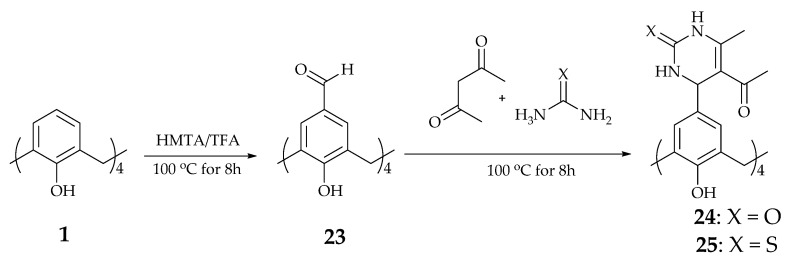
Synthetic pathway to substitute upper-rim of calix[4]arene with dihydropyrimidine [49].

There have been several reports on the methods to substitute the upper-rim and lower-rim of calix[4]arene [47,48]. Pur et al. came up with an idea to substitute the upper-rim of calix[4]arene with dihydropyrimidine moiety, as shown in Scheme 2 [49]. They devised a method to substitute four dihydropyrimidine moieties at the upper-rim of calix[4]arene by facial green Biginelli reaction. The resulting clusters of calix[4]arene-based dihydropyrimidines (**24**, **25**) showed strong antiradical activity compared to the corresponding monomers.

**Figure 5:**
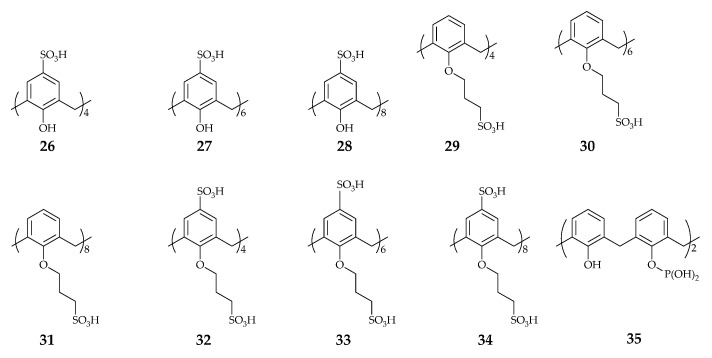
Structures of calix[4]arene derivatives **26**–**35**.

Recently, Stephens et al. reported on the structural requirements for the antioxidant activity of calix[n]arenes and their associated antibacterial activity [50]. In this work, they studied ten calix[n]arene derivatives **26**–**35** for their antioxidant and antibacterial activities by linking them to Ag nanoparticles (Figure 5). In this study, they found that the calix[n]arenes bearing sulphonate groups on the upper-rim and/or lower-rim have intrinsic antioxidant capacity. The less sulfonated calix[n]arenes necessitate the linkage to Ag nanoparticles in order to achieve similar efficacy. Therefore, these results indicate that the calix[n]arenes may be used as therapeutic agents in several diseases related to oxidative stress.

**Figure 6:**
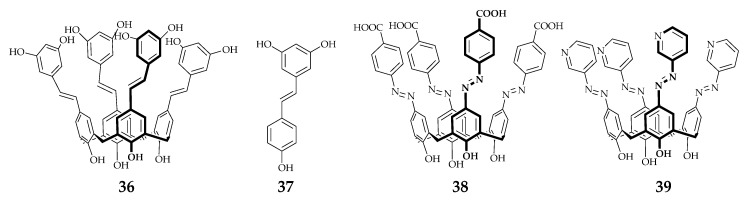
Structures of calix[4]arene derivatives **36**–**39**.

Lu et al. reported the theoretical study that allowed one to find a way for improving the antioxidative activity of resveratrol by calix[4]arene-like tetramer [51]. In this study, they designed tetra-(3,5-dihydroxy)styryl-calix[4]arene (36, a calix[4]arene-like tetramer of resveratrol) and theoretically studied at the DFT-BP86/6-311+G(d,p) level of theory for antioxidant activity in comparison to resveratrol **37** (Figure 6). The spin density in 4′-phenoxyl radicals and cation radicals are found to delocalize over the whole molecular skeleton of **36**, which allows one to form more stable radicals than that of resveratrol. Further, it was revealed that calix[4]arene scaffold plays a crucial role in the delocalization of the unpaired electron. Therefore, the synthesis of analogues derived from the conjugation of calix[4]arene and resveratrol derivatives can lead to potentially strong antioxidant molecules.

Recently, Ni et al. reported the antiradical and antioxidative activity of azocalix[4]arene derivatives **38**, **39** (Figure 6) [52]. In this work, they developed two azocalix[4]arene derivatives by a diazo coupling reaction between calix[4]arene and diazonium salts. Their antiradical and antioxidative activity of these compounds was evaluated by tow assays (hydroxyl radical scavenging assay and the pyrogallol autoxidation inhibition assay). The experimental and theoretical results identified both compounds **38** and **39** as potent antiradical and antioxidant agents. The synergism between the calix[4]arene moiety and the *para*-azo substituent groups at the upper rim contributed significantly towards the antiradical and antioxidant activity.

**Scheme 3:**
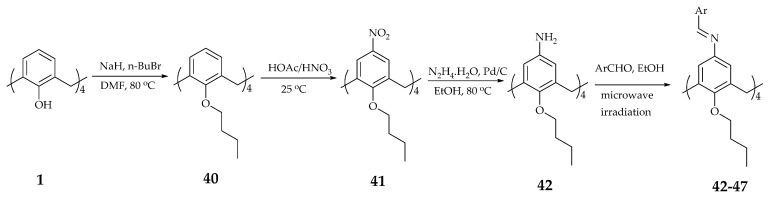
Synthetic pathway for various derivatives of iminecalix[4]arenes [55].

**Figure 7:**
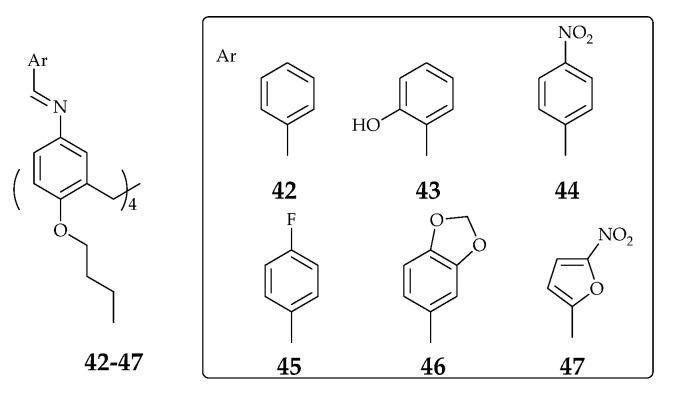
Structures of iminecalix[4]arene derivatives **42**–**47**.

Iminecalix[4]arene derivatives are known to possess a deep cavity and hyperconjugation [53]. The water-soluble iminecalix[4]arene derivatives are reported for the molecular recognition of various organic molecules [54]. However, only recently, Silva et al. reported the novel iminecalix[4]arene derivatives (**42**–**47**) with anticandidal activity (Scheme 3) [55]. However, there are no reports on the antioxidant activity of iminecalixarene derivatives. This class of calixarene has a long *pi*-aromatic conjugation system, which with proper substitutions, can be exploited for the antioxidant activities (Figure 7).

Recently, Ozgu et al. reported on the iminecalix[4]arenes containing sulphonamide moieties [56]. The novel calix[4]azacrown sulphonamide Schiff bases were synthesized by the condensation of calix[4]azacrown aldehydes with various primary and secondary sulphonamides. The obtained compounds demonstrated relevant antioxidant activity compared with standard antioxidants used in the study.

### 2.2. Resorcinarene (calix[4]resorcinarene)

**Scheme 4:**
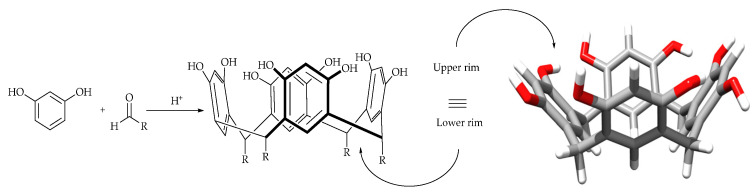
General scheme for the synthesis of resorcinarene derivatives.

Resorcinarenes are a type of calixarene obtained by the condensation of 1,3-dihydroxybenzene (Resorcinol) with an aldehyde in an acidic environment (Scheme 4) [57,58]. Similar to calix[4]arenes, resorcinarenes also assume a cone shape, with eight hydroxyl groups on the upper-rim and four alkyl groups on the lower-rim [59,60]. Resorcinarene has been extensively studied for various applications along the lines of host-guest chemistry and redox-active macrocycles [61,62,63]. Since resorcinarene is a polyhydroxy-supramolecule, it offers a highly efficient scaffold for the development of antioxidants that can be tailored by varying the functional groups. Stable cyclic nitroxides are known to possess remarkable antioxidant activity due to their ability to scavenge superoxide, peroxide, and alkyl radicals [64,65,66].

**Figure 8:**
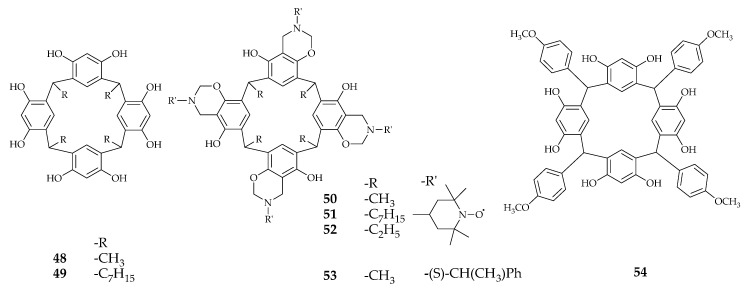
Structures of resorcinarene derivatives **48**–**54**.

**Scheme 5:**
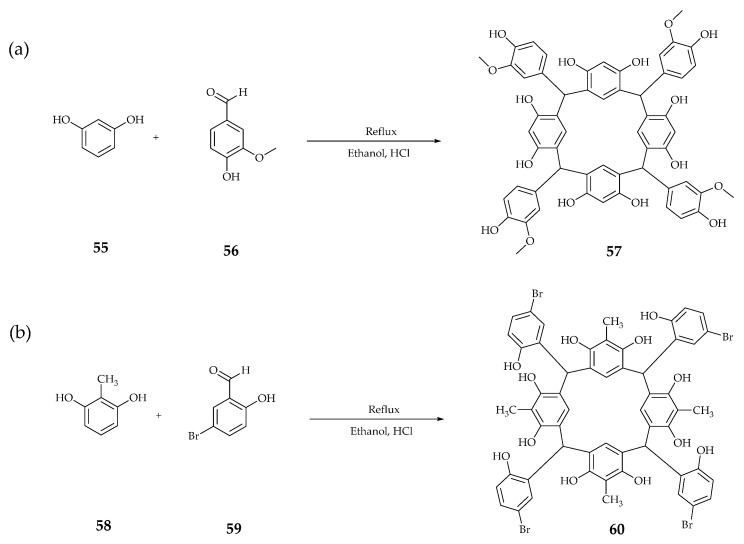
General scheme for the synthesis of resorcinarene derivative (**a**) compound **57** containing vanillin arms; (**b**) compound **60** containing 5-bromo-2-hydroxybenzene arms.

In 2009, Vovk et al. reported on the antioxidant and antiradical activities of resorcinarene tetranitroxides (Figure 8) [67]. They synthesized the resorcinarene (**48**, **49**) derivatives (**50**–**53**) by the aminomethylation of resorcinarene octols with 4-amino-TEMPO TEMPO ((4-Amino-2,2,6,6-tetramethylpiperidine-1-oxyl), and studied their antioxidant activities. They found that the Tetra- TEMPO resorcinarenes (**50**–**53**) are efficient scavengers of DPPH radicals and showed superoxide dismutase-like activity. These compounds were also found to have a high efficiency for inhibition of ABAP-induced peroxidation of linoleic acid. The macrocyclic structure of resorcinarene and intramolecular hydrogen bonding had a considerable contribution to the antiradical activity of these compounds. Recently, Ngurah reported the antioxidant activity of C-methoxyphenyl calix[4]resorcinarene **54** (Figure 8) [68]. The compound **56** showed reasonable antioxidant activity (IC50 = 79 ppm), but less than that of vitamin C (IC50 = 20.96 ppm), which was used as a standard in this study. Oliveira et al. reported the synthesis and comparative study on the antioxidant and anti-toxoplasma activities of vanillin **56** and its resorcinarene derivative **57** (Scheme 5a) [69].

The in vivo acute toxicity assays of **56** and **57** demonstrated a substantial safety margin indicated by lack toxicity up to 300 mg/kg during 14 days after administration. The obtained compound **57** exhibited more significant antioxidant activity (84.9%) as compared to vanillin **56** (19.4%). These results indicate that the resorcinarene has excellent potential for the development of antioxidant and antiradical compounds. Recently, Abosadiya et al. reported the synthesis, characterization, x-ray structure, and biological activities of c-5-bromo-2-hydroxyphenylcalix[4]-2-methyl resorcinarene **60** (Scheme 5b) [70]. The compound **60** was obtained by the condensation of 5-bromo-2-hydroxybenzaldehyde and 2-methylresorcinol in the presence of concentrated HCl. The compound **60** showed a strong activity (84.95%) in scavenging the DPPH free radicals. Since compound **60** is a polyphenolic compound, it inhibited the oxidation of DPPH, preferably using the hydrogen atoms to form the stable non-radical form of DPPH indicated by the formation of a pale-yellow color.

### 2.3. Calixtyrosol

Tyrosol is an essential antioxidant molecule with various biological activities. There has been an increasing interest in the development of strategies for the clustering of single drug units in order to design novel drugs. It is understood that the cluster effect can increase the pharmacological effect of a drug to a single drug unit [71].

**Scheme 6:**
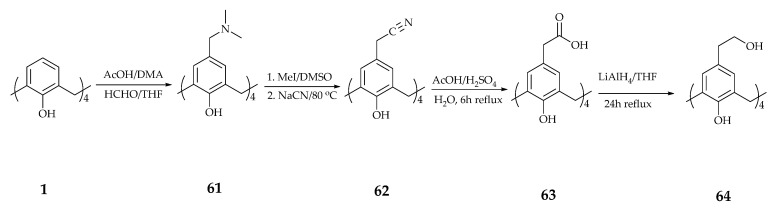
General scheme for the synthesis of calixtyrosol.

Keeping this in mind, Pur et al. developed a novel calixtyrosol **64**, which is a novel cluster of tyrosol (Scheme 6) [72]. The free radical scavenging assay demonstrated that the calixtyrosol **64** has superior antioxidant activity (>5 fold) as compared to the single tyrosol unit. The enhanced antioxidant activity is attributed to the cluster of four impacted tyrosol units that show a synergistic effect in interactions with radicals.

### 2.4. Calix[4]pyrrole

**Scheme 7:**
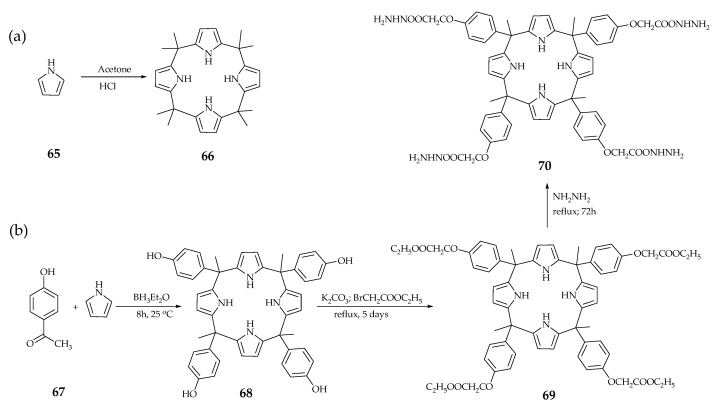
(**a**) General scheme for the synthesis of calix[4]pyrrole **66**; (**b**) Scheme for the synthesis of calix[4]pyrrole tetrahydrazide **70**.

Calix[4]pyrroles have attracted substantial attention as supramolecular containers during the past two decades [73,74]. The calix[4]pyrroles are obtained by the condensation of pyrrole with acetone in the presence of an acid (Scheme 7a) [75]. Therefore, based on the use of ketone, the reaction with pyrrole, various derivatives of calix[4]pyrroles can be synthesized (Scheme 7b) by slight modifications in the reaction conditions [76].

Even though the calix[4]pyrrole offers an excellent scaffold that can be tailored for the development of potent antioxidants, it has been understudied in this regard. However, the complex of Au and calix[4]pyrrole has been studied for the antioxidant and radical scavenging efficiencies. Kongor et al. reported the synthesis and modeling of calix[4]pyrrole derivative **70** wrapped Au nanoprobes for the detection of Pb(II), antioxidant, and radical scavenging activities [77]. The calix[4]pyrrole wrapped Au nanoprobes were found to scavenge 76.4% of DPPH free radicles. They also found that the calix[4]pyrrole 70 had low antioxidant activity in comparison to its complex with the Au. More study on the development of antioxidants based on calix[4]pyrrole scaffold is required.

### 2.5. Cucurbituril (CB)

**Scheme 8:**
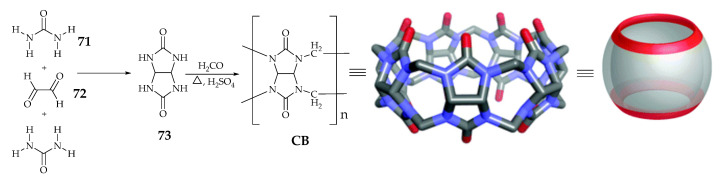
General scheme for the synthesis of cucurbituril (CB).

Cucurbituril, denoted as cucurbit[n]uril, is a supramolecule obtained by the condensation of glycoluril **73** with formaldehyde in the presence of strong acid [78]. The macrocycle was named cucurbituril, owing to its similarity to a pumpkin (*cucurbitaceae* family). The cucurbituril is often abbreviated as CB[n], highlighting the n glycoluril building blocks in the macrocycle [79,80] (Scheme 8).

**Figure 9:**
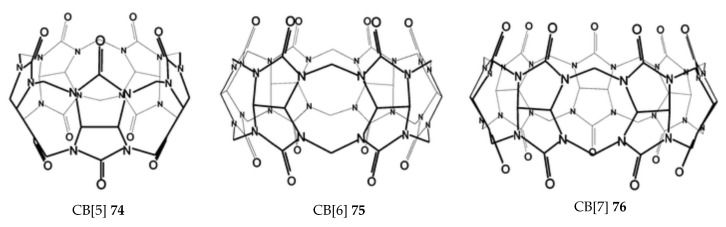
Structures of cucurbit[n]uril derivatives **74**–**76**.

Depending on the number of glycoluril units, there are various derivatives of cucurbit[n]uril, including CB[5] **74**, CB[6] **75**, CB[7] **76**, CB[8], CB[10], CB[13], CB[14], CB[15] [81,82,83] (Figure 9). Cucurbiturils have been studied for a variety of applications in chemistry, biology, and drug delivery [84].

**Figure 10:**
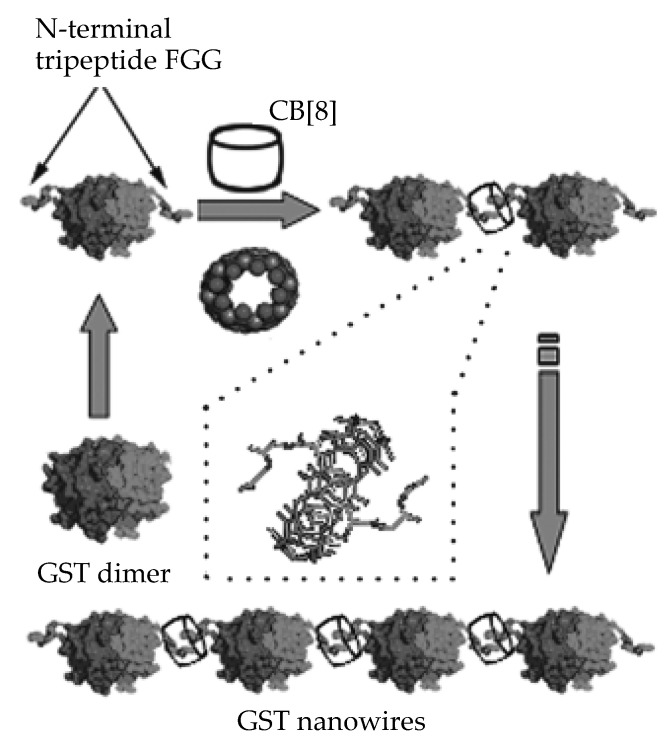
Formation of protein nanowires by CB[8]-based host–guest interactions.

Due to the excellent guest-binding ability of cucurbiturils in aqueous solutions, they play a significant role in the development of supramolecular nanostructures [85]. In 2013, Hou et al. constructed protein nanowires (Figure 10) through cucurbit[8]uril-based highly specific host-guest interaction [86]. The highly specific noncovalent interactions of CB[8] and a tripeptide FGG-tag, which was attached to the N-termini of dimeric glutathione S-transferase (GST), resulted in the formation of self-assembled protein nanowires. Then, the active site of selenoenzyme glutathione peroxidase (GPx) was introduced into GST for the construction of the functional protein nanowires. The functional protein nanowires that mimic the GPx-like activity showed high stability and exhibited substantial antioxidative properties to protect the mitochondria from oxidative stress. Therefore, the combination of cucurbituril host-guest interactions with proteins and enzymes presents a powerful tool for the construction of biologically active protein nanostructures.

**Figure 11:**
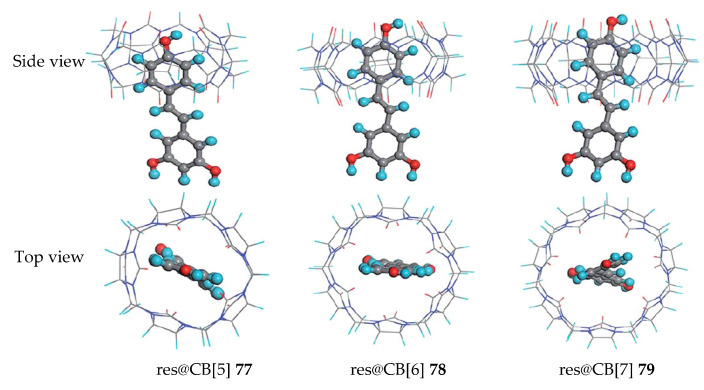
Inclusion complexes of resveratrol in cucurbiturils [87].

Since cucurbituril has been extensively studied for its host-guest chemistry, it was used for the enhancement of the antioxidant activity of known antioxidants such as resveratrol. Recently, Lu et al. reported on the use of cucurbiturils to form inclusion complexes with resveratrol (Figure 11), in order to enhance the radical scavenging activity of the resveratrol [87]. Generally, the phenolic antioxidants resveratrol demonstrate their radical scavenging activity based on three mechanisms, such as the H atom transfer (HAT) mechanism, sequential proton loss electron transfer (SPLET) mechanism, and single electron transfer (SET) mechanism. Lu et al. found that the inclusion complexes res@CB[5] **77**, res@CB[5] **78**, and res@CB[5] **79** show the enhanced antioxidant capacity of resveratrol (Figure 11).

**Figure 12:**
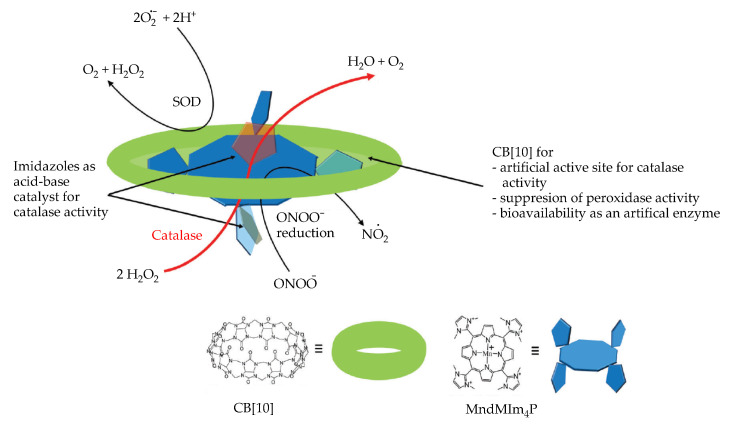
A new class of artificial enzyme (MndMIm4P@CB[10];Im) for the development of a therapeutic antioxidant [88].

Recently, Kubota et al. developed a new class of artificial enzyme composed of cucurbit[10]uril, Mn-porphyrin, and imidazole [88]. They investigated a new class of artificial enzymes obtained by simply mixing the Mn-porphyrin, imidazole, and CB[10] in the aqueous solution. In this research study, they selected Mn(III)-5,10,15,20-tetrakis(1,3-dimethylimidazolium-2-yl)porphyrin (MndMIm4P) as a novel guest for CB[10]. Their simplified process has a great advantage over earlier reports that required a multi-step synthesis for the generation of artificial catalases. The preliminary in vitro study in human cell lines suggested that the constructed artificial enzyme (Figure 12) catalytically eliminated the ROS, including H_2_O_2_. The use of CB[10] in the construction of this artificial enzyme was crucial to increase its bioavailability. This study is one of a kind that demonstrates the meticulous use of supramolecules has a high potential to generate novel molecules as therapeutic antioxidants.

### 2.6. Porphyrin

**Scheme 9:**
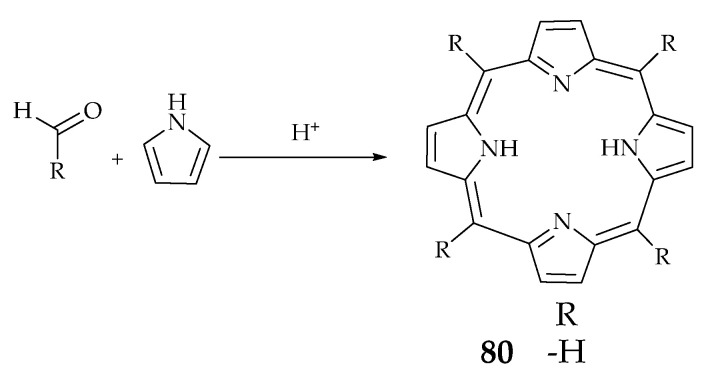
Scheme for the synthesis of porphyrin.

Porphyrins, metalloporphyrins, and related tetrapyrrolic macrocyclic compounds are a class of important heterocycles due to their wide applications in chemistry and biology [89,90]. They have been employed successfully in the areas of catalysis, medicine, and material science [91]. As shown in Scheme 9, a one-pot condensation of an aldehyde with pyrrole in acidic conditions can generate various derivatives of porphyrin **80**. Mackensen et al. reported the neuroprotection from delayed postischemic administration of a metalloporphyrin catalytic antioxidant [92]. They found that the administration of a metalloporphyrin had a significant neuroprotective effect, as it decreases postischemic superoxide mediated oxidative stress.

**Figure 13:**
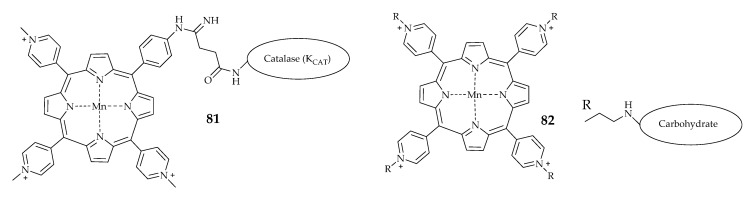
Structures of Mn-porphyrin (MnP) conjugates **81**, **82**.

Asayama et al. reported the manganese porphyrins (**81**, **82**) functionalized with the biomolecules (Figure 13) [93]. The developed porphyrin derivatives were studied for a potential role as antioxidants. The Mn-porphyrin (MnP) conjugated to catalase (**81**) was evaluated for its ability to catalyze the reduction of H_2_O_2_ to H_2_O. This derivative was found to possess a dual function as SOD and catalase. The carbohydrate conjugated MnP (**82**) facilitated the anchoring of the conjugate on the cell surface by binding to a receptor. The results of this study suggest that the porphyrins can be modified to generate analogues with potent antioxidant activities.

**Figure 14:**
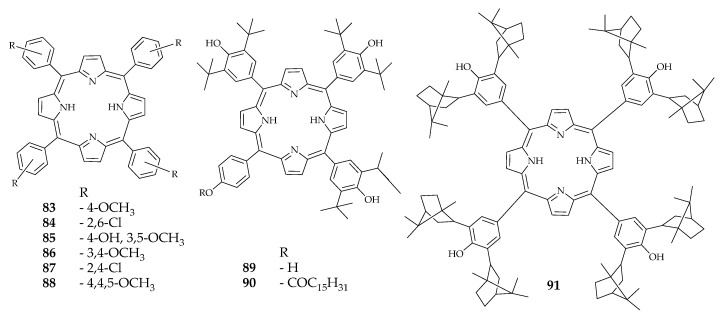
Structures of porphyrin derivatives **83**–**91**.

Fadda et al. reported the pharmacological activities of novel porphyrin derivatives [94,95]. They used a capping mechanism to produce a series of porphyrin derivatives **83**–**88** (Figure 14). All compounds were found to inhibit the peroxidation reactions in rat brain and kidney homogenates and rat erythrocyte hemolysis. The results of the ABTS assay indicated that the compounds **86** and **88** have a robust antioxidant activity, indicated by the 74.3% and 79.5% ABTS inhibition, respectively.

In 2013, Tyurin et al. reported the redox characteristics and antioxidant activity of porphyrins with 2,6-dialkylphenol groups **89**–**91** (Figure 14) [96]. All compounds demonstrated significant DPPH scavenging activity. The antioxidant activity was attributed to the readily delocalized unpaired electron, resulting in the increased stability of a product after reaction with the radical.

**Scheme 10:**
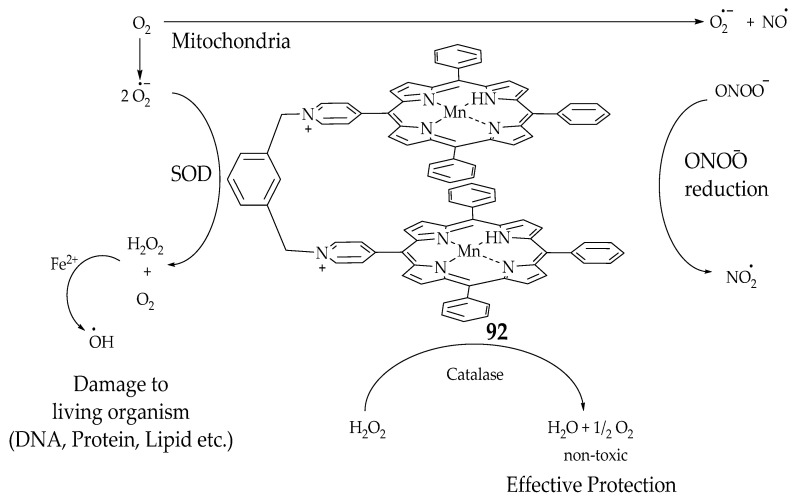
Multiple antioxidant activities of water-soluble di-nuclear Mn-Porphyrin.

Kubota et al. reported a synthesis of water-soluble MnP with antioxidant activities [97]. The Mn-porphyrin derivative **92** had a fair water-solubility and possessed SOD-like catalase-like activity. As shown in Scheme 10, the mechanistic study indicated the synergism of two Mn active sites gives a catalase-like activity to the compound. The restoration of the treadmill-running ability of SOD-deficient mouse indicates that the derivative **92** exhibited strong antioxidative activity in vivo.

**Figure 15:**
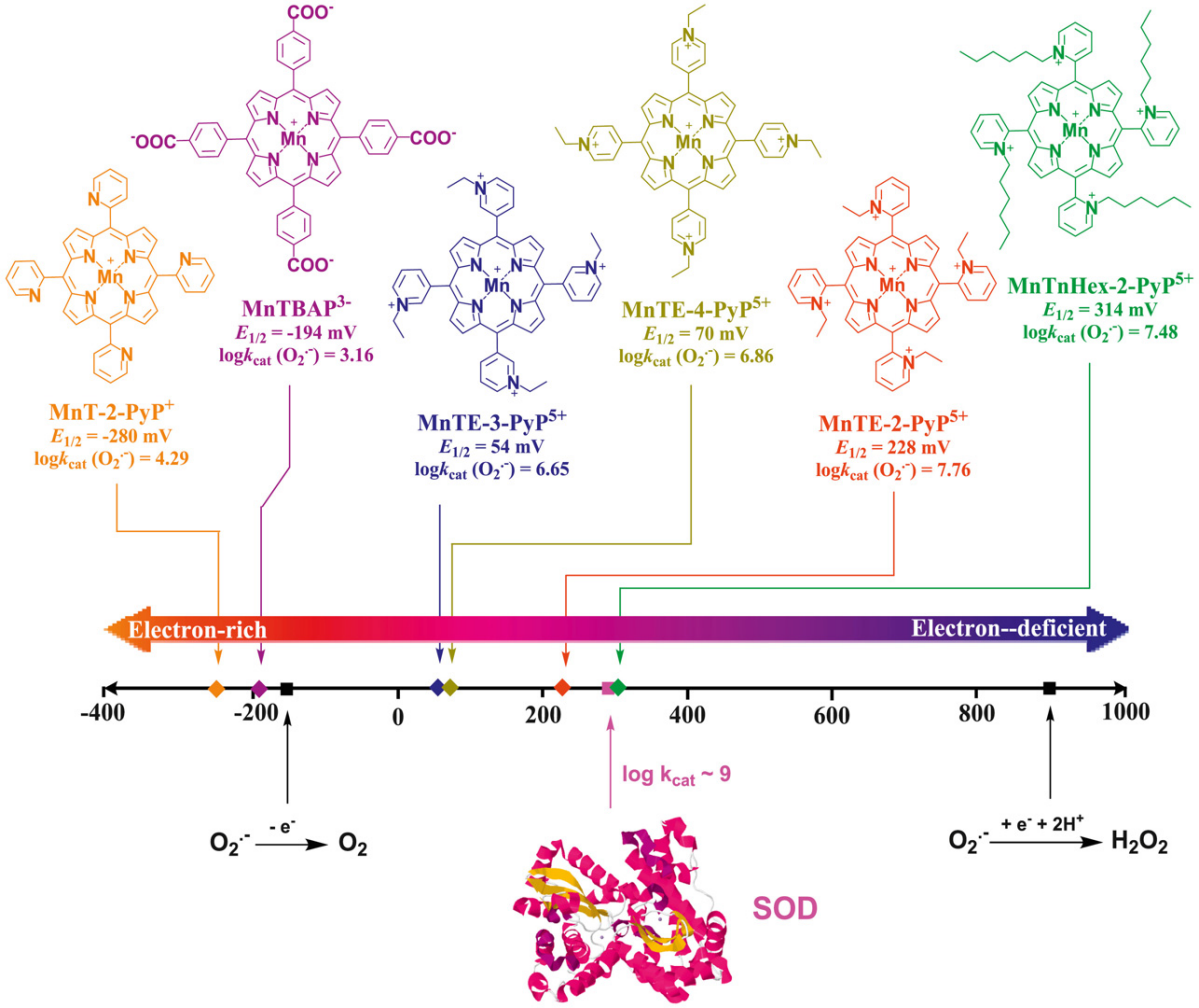
Mn porphyrins that mimic the SOD [98].

Batini-Haberle et al. reviewed the SOD mimicking the activity of Mn porphyrins [98]. As shown in Figure 15, the dismutation process involves of two steps (i) SOD enzyme or MnP-based molecule acts as a (pro)oxidant in a first step, and as an antioxidant in the second step and closes the catalytic cycle, and (ii) acting as an oxidant, they produce H_2_O_2_ in the second step. Therefore, similar to SOD, its mimic MnPs can be considered to have applications in antioxidative defense in physiological conditions.

**Figure 16:**
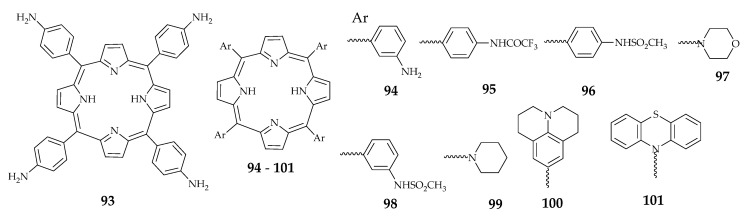
Structures of porphyrin derivatives **93**–**101**.

Tesakova et al. reported the tetraphenylporphyrene (**93**) and evaluation of its antioxidant activity by electrochemistry [99]. Abu-Melha reported the synthesis of *meso*-substituted porphyrins **94**–**101** and the evaluation of their antioxidant activity (Figure 16) [100]. Among derivatives **94**–**101**, compounds **96**, **98** and **101** exhibited the strongest antioxidant activity. The strong radical-scavenging activity of compounds **96** and **98** was credited to the presence of N and S atoms in their structures. It is well known that the presence of sulfur in compounds improves their radical-scavenging activity [101].

**Figure 17:**
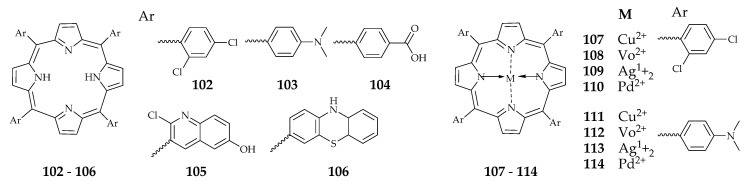
Structures of porphyrin derivatives **102**–**114**.

Porphyrins can be tailored to desired physicochemical properties by complexing with different metal ions, such as manganese, copper, zinc, silver, nickel etc. [102,103]. The *meso*-substituted porphyrins have gained considerable interest due to their ability to act as ligands for metal ions forming the complexes that have high applicability for therapeutic purposes [104,105]. Recently, Ahmed et al. synthesized and evaluated the antioxidant and anticancer activities of porphyrin derivatives **102**–**106** and their metalloporphyrin counterparts **107**–**114** (Figure 17) [106]. All compounds showed radical-scavenging activity. However, in particular, compounds **105** and **111** showed exceptional antioxidant activity as compared to ascorbic acid, which was used as a reference.

## 3. Discussion

ROS are highly reactive molecules that, in many cases, function as regulators of critical signaling pathways in living cells. The moderate levels of ROS are essential for various cellular processes, including gene expression. The elevated ROS levels alter the cell’s microenvironment and damage the several biomolecules, including proteins, DNA leading to severe diseases like cancer and CVD. The generation of ROS and other free radicals in living cells is an inevitable consequence of aerobic metabolism. The levels of these highly reactive molecules is controlled by various antioxidant enzymatic and non-enzymatic pathways in the same cells. However, as evident from the disorders as a result of dysregulation of levels of ROS, the therapeutic use of external ROS modulators is inevitable.

In the last decade, the development of radical scavengers that mimic the activities of cellular enzymes such as SOD, catalase etc. has gained a tremendous interest of various scientific communities. The compounds that mimic enzyme activity function by one of the two methods, (i) by favoring a disproportionation process (SOD, catalase mimics) (ii) by undergoing reduction during the oxidation reactions (glutathione peroxidase mimics). There has been a tremendous amount of research on the identification of antioxidants from various natural sources. However, the development of synthetic antioxidants for therapeutic applications is a comparatively understudied field. The design, development, and synthesis of highly functional molecules with strong radical-scavenging activity warrants a suitable molecular scaffold that can be tailored for required physical, chemical, and biological properties.

Supramolecules such as calix[n]arene, resorcinarene, calixtyrosol, calixpyrrole, cucurbit[n]uril, and porphyrin were used for the development of radical scavengers (Table 1). Calix[n]arens are the excellent scaffolds for the design of synthetic antioxidants, as the upper and lower rims of these molecules can be readily transformed by using various chemical reactions. The calix[n]arenes show a considerable amount of radical scavenging ability, owing to the aromatic hydroxyl groups that help in quenching the free radicals. Similar to the resveratrol derived analogues of calix[4]arenes, several other known antioxidant moieties can be substituted on the calixarene scaffold to afford stronger radical-scavengers. The resorcinarenes, as discussed earlier, can be used for the synthesis of functional compounds that may have significant applications in redox biology. The resorcinarene derivatives are efficient DPPH radical scavengers and were found to have SOD-like activity. The ability of resorcinarene to quench the free radicals is attributed to the intramolecular hydrogen bonding of these compounds. Calixtyrosol and calixpyrrole derived compounds were found to be excellent ROS modulators. However, these classes of macromolecules warrant further investigation in the development of therapeutically useful antioxidant compounds. The cucurbituril scaffold has been used for the development of molecular containers for various applications, including the enhancement of the radical-scavenger abilities of known antioxidants. The inclusion complexes of cucurbituril with molecules like resveratrol already proved beneficial to increase the reductive power of resveratrol and similar compounds.

Furthermore, the enormous study on the reaction mechanisms for the generation of novel derivatives, the cucurbituril scaffold, can be fine-tuned for the desired purposes. Hence, to see the novel antioxidants based on the cucurbituril scaffold in the near future is highly awaited. Among all supramolecules described in this review, porphyrins and their metal-complexed analogues are studied extensively; both in vitro and in vivo experiments for the radical-scavenging activities. As mentioned earlier, the porphyrin derivatives mimic the SOD and catalase like activities and thus have a great potential to be used therapeutically.

## 4. Conclusions

In conclusion, supramolecular chemistry offers several classes of macrocycles, including calix[n]arene, resorcinarene, calixtyrosol, calixpyrrole, cucurbit[n]uril, and porphyrin. There are several reports on the modification reactions of these molecules to product functional compounds of desired properties. However, the application of these macromolecular scaffolds for the development of potent radical scavengers with pharmacological activities are not studied to its full potential. Therefore, this review elaborated on the derivatives of various supramolecules and their antioxidant activities. Porphyrin derivatives have a significant amount of research on their radical-scavenging abilities and reaction mechanisms supporting them. Similar to porphyrins, the calix[n]arene, resorcinarene, calixtyrosol, calixpyrrole, and cucurbit[n]uril derivatives offer great potential for the development of ROS modulators with significant pharmacological activities to prevent and treat the disorders resulting from oxidative stress.

## Figures and Tables

**Table 1:** Antioxidant activity of selected derivatives of calix[n]arene, resorcinarene, calixtyrosol, calixpyrrole, cucurbit[n]uril, and porphyrin.

Supramolecular Scaffold	Compound	DPPH Radical Scavenging (%)	*k_inh_*/*k_p_* (M^−1^ S^−1^) ^a^	*k_SOD_* Activity (M^−1^ s^−1^)	Ref.
Calix[n]arene	7	-	7.25 × 10^5^	-	[44]
	8	-	5.75 × 10^5^	-	
	18	82.6	-	-	[45]
	38	^b^ 71.7	-		[52]
	39	^b^ 63.2	-		
Resorcinarene	50	^c^ 0.19 ± 0.04	-	-	[67]
	52	^c^ 0.18 ± 0.01	-	-	
	54	86.3	-	-	
	57	84.9	-	-	[70]
	60	85.0	-	-	
Calixtyrosol	64	Significant	-	-	[72]
Calixpyrrole	70	76.4%	-	-	[77]
Cucurbit[n]uril	77–79	^d^ Significant	-	-	[87]
	MndMIm4P@CB[10];Im	-	-	1 × 10^7^	[88]
Porphyrin	80	-	-	5.4 × 10^5^	[92]
	86	74.3	-	-	[95]
	88	79.5	-	-	
	90	77.36	-	-	[96]
	113	83.40	-	-	[106]

^a^*k_inh_* and *k_p_*—rate constant of inhibition of free radical and propagation rate constant of the chain reaction, respectively. ^b^ [^•^OH] radical scavenging (%) activity. DPPH = 2,2-diphenyl-1-picrylhydrazyl radical. ^c^ ECR_50_ = [scavenger]/[DPPH] producing 50% scavenging of DPPH after 5min reaction time. ^d^ antioxidant activity was higher than resveratrol.
